# Microscopic Identification of Anatomical Elements and Chemical Analysis of Secondary Nests of *Vespa velutina nigrithorax* du Buyson

**DOI:** 10.3390/insects13060537

**Published:** 2022-06-10

**Authors:** Nazaret Crespo, José Louzada, Lisete S. Fernandes, Pedro B. Tavares, José Aranha

**Affiliations:** 1 Department of Forestry Sciences and Landscape Architecture (CIFAP), University of Trás-os-Montes and Alto Douro, 5000-801 Vila Real, Portugal; jlousada@utad.pt (J.L.); j.aranha.utad@gmail.com (J.A.); 2 Centre for the Research and Technology of Agro-Environmental and Biological Sciences (CITAB, Inov4Agro), University of Trás-os-Montes and Alto Douro (UTAD), Quinta de Prados, 5000-801 Vila Real, Portugal; 3 Chemistry Department, University of Trás-os-Montes and Alto Douro, 5000-801 Vila Real, Portugal; ume@utad.pt (L.S.F.); ptavares@utad.pt (P.B.T.); 4 Electron Microscopy Unit (CIDE-UME), University of Trás-os-Montes and Alto Douro, 5001-801 Vila Real, Portugal

**Keywords:** anatomical elements, nest, *Vespa velutina* *nigrithorax*, X-ray diffraction (XRD), scanning electron microscopy (SEM/EDS)

## Abstract

**Simple Summary:**

*Vespa velutina nigrithorax*, one of the eleven subspecies of *Vespa velutina*, has become a matter of concern due to its invasive behavior after its accidental introduction into France in 2004. This *Vespa* species, also known as the yellow-legged hornet, builds large nests, the structure and chemical composition of which are of interest to know and understand in order to estimate the areas that these hornets may find acceptable for establishing new colonies. Its presence causes serious negative impacts on ecosystems and on pollinating species, especially on honeybees. As the presence of this type of hornet is very recent in Europe, there are large gaps in our knowledge about its adaptability and control measures. The main objective of this study is to identify the composition of the materials used by the Asian hornet in the construction of secondary nest envelopes. The samples that were collected from each nest were analyzed using two microscopic devices in non-disaggregated and disaggregated material. The same samples were also chemically analyzed by means of X-ray diffraction and SEM/EDS (scanning electron microscopy). It was noticed that the nests were formed by the agglomeration of small particles that are spatially organized in brown and beige strips. The microscopic analysis showed the presence of cellular elements of agricultural origin, woody material from forest species, and leaves, almost all of which were lignocellulose materials. The results achieved from chemical analysis led to the conclusion that the main chemical constituent of the nests is cellulose. As for the elemental analysis, about 99% of the nests were composed of C and O and low amounts of other microelements (Na, Al, Si, K, and Ca).

**Abstract:**

*Vespa velutina* accidentally arrived in Europe (France) in 2004, and rapidly expanded throughout the entire country. Its presence in mainland Portugal was first noticed in 2011. Being an invasive species with no natural predators in the region to control it, it has caused enormous environmental and economic damage, particularly on *Apis mellifera* (honeybee) colonies. Although there is already some research on this species’ biology, little is known about its adaption to European ecological conditions, specifically in terms of nest building. This type of hornet builds a primary nest in the spring to start a colony. During the summer, they build a secondary nest to develop the main colony. These secondary nests are ovoid-shaped and range in size from 18.7 cm to 45.0 cm in diameter and from 19.2 cm to 65.0 cm in length, attaining their highest development in late summer. The external appearance of these nests is characterized by alternating stripes that are beige and brown in color. The main objective of this study is to identify the composition and the origin of the materials that are used by *Vespa velutina nigrithorax* to build the outer envelope of these secondary nests. This information could be very interesting and will not only increase our knowledge on the biology of the species in regions far from its original area, but will also be relevant for the future implementation of new policies to control this invasive species by means biological control. Several samples were taken from each nest and were observed under different optical magnifying devices. In the second stage, their chemical composition was analyzed by X-ray diffraction (XRD) and scanning electron microscopy (SEM/EDS). It was noticed that almost all of the materials used in the nests’ construction were lignocellulose from woody materials from both softwood (gymnosperm) and hardwood (angiosperm) forest species as well from leaves and small particles of agricultural origin (grasses). The beige strips were formed almost exclusively from woody softwood cells, while the brown strips were composed of hardwood cells, leaf tissues, and grasses. Chemically, it was noticed that this material mainly consisted of cellulose, with more than 99% being composed of C and O and very little mineral material from elements such as Na, Al, Si, K, and Ca. The achieved results allow us to state that in the construction of these secondary nests, these hornets only used organic materials that are then probably agglomerated through their mouths.

## 1. Introduction

The order Hymenoptera is one of the four main Orders of insects that exists in the world [1], within this Order are bees, ants, wasps (including hornets), etc., which have a similar morphology and also a wide variety of shapes and sizes [1]. Social wasps also have a great ability to build nests, which are quite complex and impressive [2].

*Vespa velutina*, Lepeletier, 1836, is a species of social wasp that is native to the tropical and subtropical regions of Southeast Asia [3,4], from northern India to the Indochina peninsula, Taiwan, and Indonesia [3]. This wasp is one of the 22 species of the genus *Vespa* that exist around the world (all of them present in Asia); this genus is divided into eleven subspecies, and *Vespa velutina nigrithorax*, du Buysson, (Asian hornet or yellow-legged hornet) is one of them [3].

In 2004, the presence of an Asian hornet in Europe was confirmed for the first time, and was first confirmed in southeastern France [5], before rapidly expanding to other parts of the country [6]. It has now become a naturalized and well-established species [7].

From 2004 to the present, it has expanded to other European countries. In 2010, its presence was noticed in Navarra (Spain) [6]; in 2011, it was noticed in Flobecq (Belgium) [7]; and in 2011, it was noticed in Viana do Castelo (Portugal) [8]. In 2012, it arrived in Liguria (Italy) [9], in Karlsruhe (Germany) in 2014 [10], and in the Balearic Islands (Spain) in 2015 [11]. In 2016, it officially announced its presence in Gloucestershire (Great Britain) [12], and in 2020, it was found in Luxembourg [13]. *Vespa velutina nigrithorax* has become the first successfully invasive Asian hornet species in Europe [4,5]. In 2014, it was classified under Regulation 1143/2014 of the European Parliament and as an invasive alien species of concern to the Union during the Council of 22 October 2014 [14].

The first sighting of *Vespa velutina nigrithorax* in Portugal occurred in September 2011 [8,15,16] in the county of Viana do Castelo (northwest Portugal). The first nests were detected a year later in the same county in trees located on the banks of the Lima River near the river mouth. This invasive hornet spread quickly to neighboring counties at an alarming rate, spreading at an estimated 13.2 km/year [15]. Some authors have demonstrated that the dispersal mechanism of this species could be based on the road network and motorways in Portugal [16].

Over the years, this social and predatory species has caused serious negative impacts on ecosystems and pollinating species [17,18], mainly on *Apis mellifera* (honeybee) colonies [4,17,19], and has consequently caused economic problems in beekeeping and fruit production [20]. Additionally, it has caused public health problems due to stings [21]. Thus, in 2014, the “Action Plan for the Vigilance and Control of the *Vespa velutina* in Portugal” was created [22], with the aim of mitigating the impact caused by the yellow-legged hornet in the areas where it is already present; avoiding new outbreaks in regions that have not yet been invaded; and preventing the its spread to other Portuguese counties, particularly to the Azores and Madeira archipelagos. This action plan was developed by the Directorate-General of Food and Veterinary (DGAV) and by the Nature and Forest Conservation Institute (ICNF), with contributions from the National Institute for Agricultural Research and Veterinary IP (INIAV) and was updated in 2018 [22].

Since the confirmation of the first *Vespa velutina nigrithorax* nest in Portugal in 2012, the University of Trás-os-Montes and Alto Douro (UTAD) has been monitoring the occurrence and dissemination of the species. Two of the most important projects in which UTAD has been involved in were: GESVESPA (“Strategies for sustainable management of *Vespa velutina* in North Portugal” (POSEUR-03-2215-FC-000008)) from 2016 to 2018 and GOVESPA (Operational Group *Vespa velutina*: Control and minimization of losses caused by the invasive species *Vespa velutina nigrithorax* (*Vespa velutina*) in beekeeping and honey production) from 2018 to 2022.

Considerable research has been conducted on *Vespa velutina nigrithorax* around the world regarding [23]: its biology [4,24,25,26,27,28,29], caste differentiation [30,31], unnatural distribution around the world [12,32,33], the most effective methods for catching hornets [34], etc. However, few studies demonstrating the true composition of the outer envelope of the nests’ of this invasive species has been presented [35,36], nor has there been much research on the anatomical elements and chemical structure of the nests of the species.

According to data provided by the Portuguese Institute for Nature Conservation and Forests (Instituto da Conservação da Natureza e das Florestas, ICNF) through the SOSvespa (2015–2020) and STOPvespa platforms (2020 to date), the first confirmed and recorded yellow-legged hornet nests in the Trás-os-Montes and Alto Douro region, were in 2018 [15]. Since then, this invasive species has been noticed in all of the counties of this Portuguese region.

Thus, the main objective of this study is to characterize each of the secondary nests of *Vespa velutina nigrithorax* that have been removed from the interior-north of Portugal as well as to identify the anatomic materials and chemical composition used by this species to build the outer envelope of the nests. This set of information not only is very important for understanding the biology of this hornet species in regions far from its area of origin but also is crucial for areas in which this species may find it acceptable to establish new colonies, allowing the establishment of a possible future policy regarding biological control and to ability to combat this invasive species.

## 2. Materials and Methods

### 2.1. Location and Characterization of the Study Area

**Figure 1:**
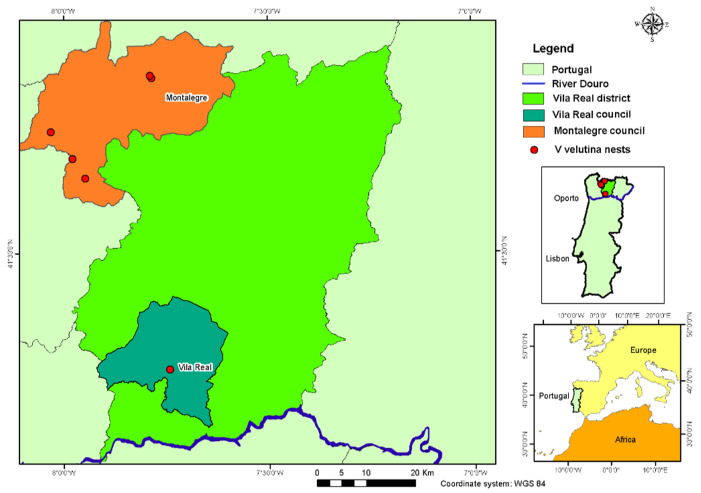
Geographic location: *Vespa velutina nigrithorax* nests.

The study region is located in Northern Portugal (Figure 1) and comprises a predominant agroforestry area of ha (1.9% urban, 42.0% agriculture, 55.3% forestry, and 0.9% water bodies). As the study area extends from the Douro River to the high mountains of Montalegre, it comprises several of the morphological and edaphoclimatic conditions that are present in this region. The altitude varies from 50 m to 1295 m, the average annual temperature varies from 7.5 °C to 16 °C, and the total annual precipitation varies from 400 mm/year to 3000 mm/year [37]. The landscape is very fragmented and is characterized by rural settlements that are surrounded by agricultural areas that are side by side with bush and forest land.

### 2.2. Nests Data

This study was carried out using six secondary nests (Table 1) of *Vespa velutina nigrithorax* (yellow-legged hornet), located in the counties of Vila Real and Montalegre (Table 2 and Figure 1). All of the nests were removed using the same method and by a specialized technician.

Five of the six nests were removed using the “tree climbing technique” to ensure better structural conservation both externally and internally and to ensure that all of the hornets and larvae remained inside the nest (especially the queen hornet). This nest removal method was carried out as follows: at night, the technician climbed the tree using a red light to see (hornets cannot see in red wave lengths), and once the nest entrance was located and reached, a biocide was applied inside the nest. Then, the nest was wrapped in a 90 L black plastic bag; the nest’s supporting tree branch was cut off; and the nest placed in a freezer at −21 °C for 2–3 days, thus ensuring the non-survival of each individual, larva, and egg. Nest002” was the only nest where climbing was not performed, as it was located at a roof structure inside of a storage house.

The nests were removed from forest species that were characteristic of the place where they were found: from softwoods (Nest001 and Nest003) and hardwoods (Nest004, Nest005, and Nest006), as presented in Table 2.

All of the nests were composed of an outer envelope thickness that was 12.0 cm to 15.0 cm on average and several internal comb platforms where the eggs and larvae are kept.

### 2.3. Microscopic Identification of Anatomical Elements

**Figure 2:**
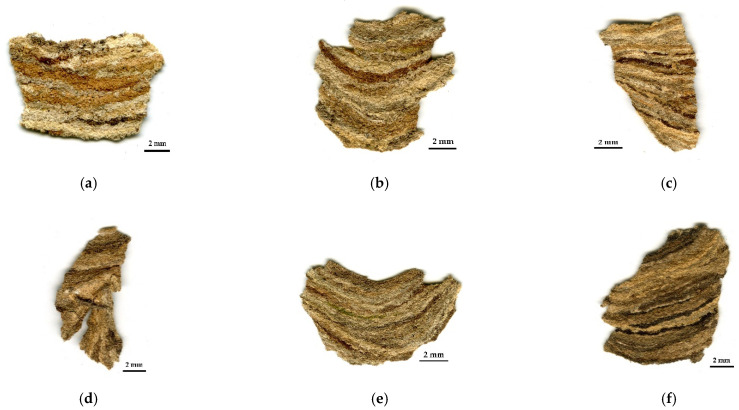
Examples of samples from the outer envelope from each nest *Vespa velutina nigrithorax*: (**a**) Nest001, (**b**) Nest 002, (**c**) Nest003, (**d**) Nest004, (**e**) Nest005, and (**f**) Nest006 (Zoom 3×).

The anatomical identification of the outer envelope elements of the secondary nests of *Vespa velutina nigrithorax* was carried out in two phases: the first phase was carried out over non-disaggregated material, and the second phase was carried out over disaggregated material. Six randomly selected samples were taken from around the nest that were 5 cm by 5 cm in size and had an average weight of approximately 1 g. From these samples, subsamples were collected from the beige strips and from the brown strips for anatomical analysis. The characterization of the strips color was perdormed by the RGB and CIELab system. From each nest sample (presented in Figure 2), one digital image was obtained by a camera (AVT Marlin F-145C2, Stadtroda, Germany) and transferred to a computer. The color analysis was performed using Image Pro-Plus 6.2 software (Media Cybernetics, Bethesda, USA), pixel by pixel, by the system RGB (Red, Green, Blue) along a line drawn in the longitudinal direction in each of the brown and beige stripes.

After evaluating the average RGB value of each of the brown and beige stripes, their conversion to the CIELab system was carried out using the Convertingcolors software (https://convertingcolors.com (accessed on 27 May 2022)).

#### 2.3.1. First stage: Intact Material (Non-Disaggregated Material) Analysis

In the first stage, the non-disaggregated material from the beige and brown strips was observed with a binocular magnifying glass (Nikon SMZ-10) with variable magnification ranging from 10× to 40× to identify the structural elements.

#### 2.3.2. Second Stage: Disaggregated Material in Distilled Water

In the second stage, the structural material from the beige and the brown strips was dipped in distilled water to disaggregate its structure into its constituent elements. The samples that were taken from each of the nests were placed into glass test tubes with distilled water in a 60° water bath at for 48 h. After this time, the sample solution was stirred with a glass rod to dissociate the elements.

#### 2.3.3. Second Stage: Disaggregated Material by the Franklin Method

In this stage, the Franklin method was used to dissociate the lignocellulosic materials. The beige and brown samples from each nest were placed into glass test tubes with a 1:1 *v/v* solution of glacial acetic acid and hydrogen peroxide at 60 °C for 48 h. Then, the samples were washed out with distilled water to completely remove the solution. Finally, the macerate material was stirred with a glass rod, not only to obtain the separation of the different particles from the samples, but also to achieve the dissociation of most of the constituent elements of the particles.

These dissociated elements from each nest, from both the brown and beige strips, were then placed in Petri dishes and glass slides to observe and identify the dissociated elements using a Nikon SMZ-10 binocular magnifier (10× to 40× magnification), a Baty-Shadomaster SM 20 inverted microscope (50× and 100× magnification), and an Olympus BX 50 fluorescence microscope (240× magnification).

### 2.4. Chemical Analysis

#### 2.4.1. X-ray Diffraction (XRD)

X-ray diffraction (XRD) data were collected at room temperature using a PANalytical X’Pert Pro diffractometer equipped with an X’Celerator detector and a secondary monochromator with θ/2θ Bragg-Brentano geometry. Measurements were performed using 40 kV and 30 mA, CuKα radiation (λα1 = 1.54060 Å and λα2 = 1.54443 Å), a 0.017°/step, and a 100 s/step at an angular range of 7–80° 2θ. The phases that were obtained were identified using HighScore Plus 4.8 software, version 4.0, Malvern Panalytical, Malvern, UK.

#### 2.4.2. Scanning Electron Microscopy (SEM) Analysis with Energy Dispersive Spectroscopy (EDS)

The chemical analysis was complemented with a scanning electron microscopy (SEM) study since this technique, being more precise, enables the identification of not only the main chemical elements that are present in the material, but also other microelements that are present in very small amounts. This was performed to deepen our knowledge about the chemical composition of *Vespa velutina nigrithorax* nests.

Samples from the beige and the brown strips were placed into aluminum stubs and fixed with carbon tape as a way to prepare them for the scanning electron microscopy (SEM) study (SEM/ESEM FEI QUANTA–400).

A low vacuum mode was used for visualization, with the partial pressure inside the chamber being 1.30 mbar and an acceleration voltage of 20 kV. Several photographs were taken at different resolutions.

Energy dispersive spectroscopy (EDS/EDX) equipment was used for the chemical analyses, and the same acquisition time was used for all of the different samples.

### 2.5. Statistical Analysis

The values that were achieved for the chemical composition of the nests were submitted to Tukey’s multiple range test, with significance being set to 95%, and to a one-way ANOVA to check if the compositions of the different strips on the nests (beige and brown) were different from each other. All of the statistical analysis was performed using the JMP statistical software package (SAS Institute).

## 3. Results

### 3.1. First Stage: Intact Material (Non-Disaggregated Material)

Macroscopic observations of the nest samples, shown in Figure 2, revealed that they were formed by the agglomeration of small particles (flakes) and organized in parallel strips with different shades of brown, gray, and, occasionally, green punctuation, regardless of the original location of the nest in nature.

However, although this visual analysis indicates that the flakes used during nest construction originate from lignocellulose materials (Figure 2), it was not possible to conclude the origin of these vegetal materials. Thus, it was necessary to continue the study through the microscopic analysis of the disaggregated material.

### 3.2. Second Stage: Disaggregated Material Analysis

Using an inverted microscope, it was possible to identify and to measure some of the cellular elements present in the substrate, namely, wood cellulose from forest species, both softwoods and hardwoods, as well as materials of agricultural origin, namely, grass plants.

**Figure 3:**
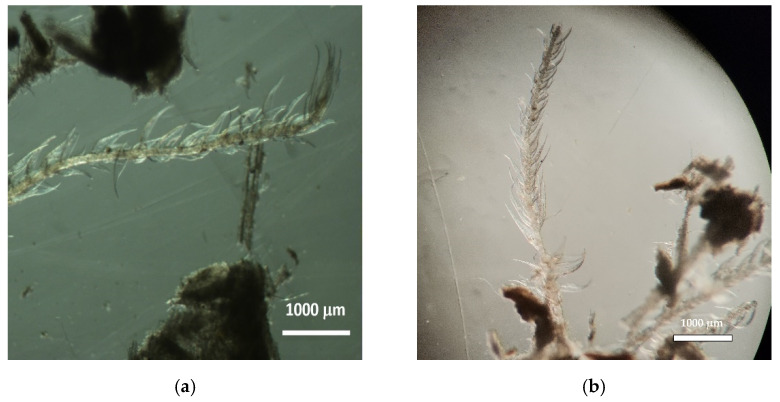
Images of cellular structures of agricultural origin (grasses): (**a**) Nest006 and (**b**) Nest004 (Zoom 10×).

**Figure 4:**
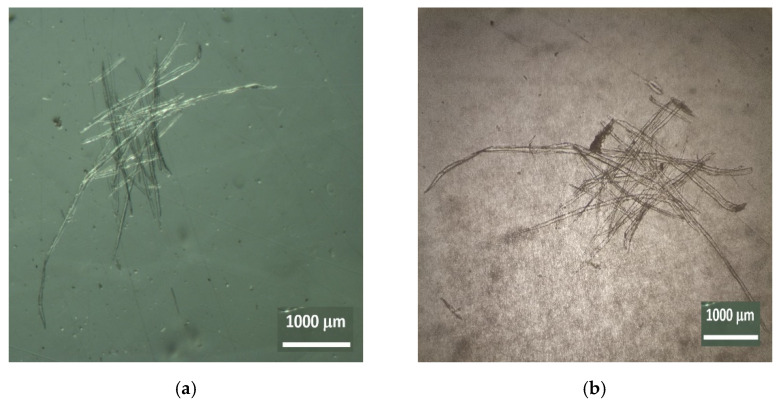
Tracheid cells of a softwood (gymnosperms) species (**a**) Nest006 and (**b**) Nest005 (Zoom 10×).

The cellular structures of agricultural origin (grasses) are shown in Figure 3a,b, and the tracheid cells of a softwood (gymnosperms) species are shown in Figure 4a,b.

**Figure 5:**
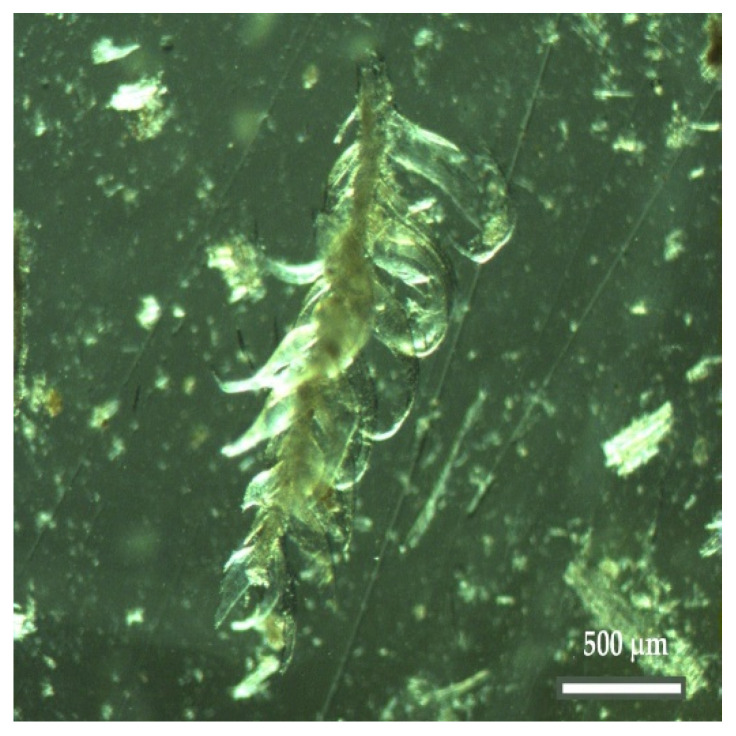
Vegetal material of agricultural origin in Nest006 (Zoom 20×).

**Figure 6:**
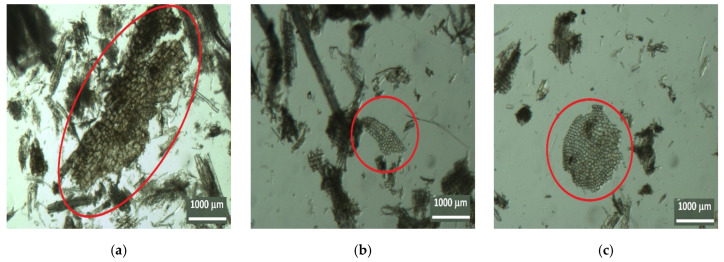
Parenchyma cells of the leaf structure ((**a**) Nest003, (**b**) Nest005, and (**c**) Nest005) (Zoom 10×).

**Figure 7:**
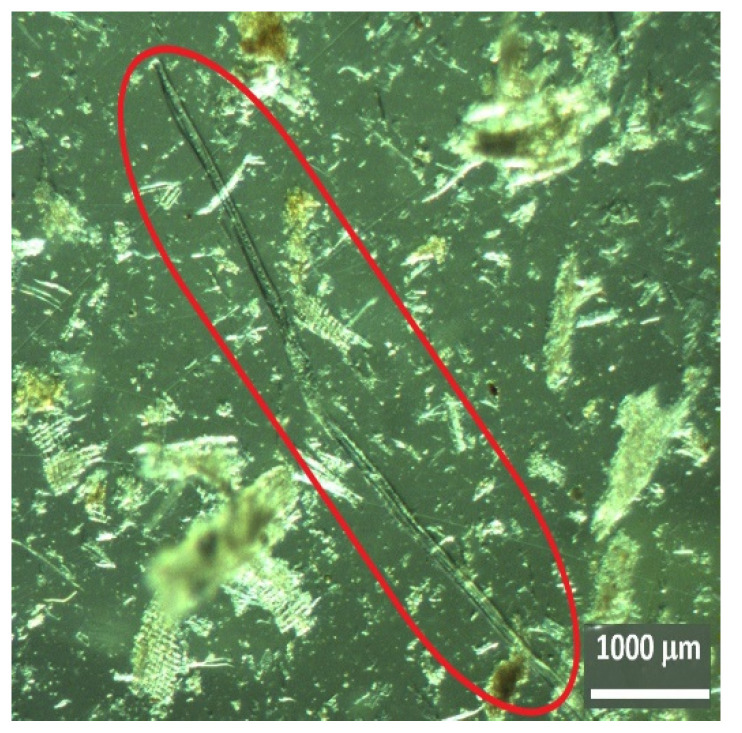
Tracheid cells with bordered pits (Nest006) (Zoom 10×).

**Figure 8:**
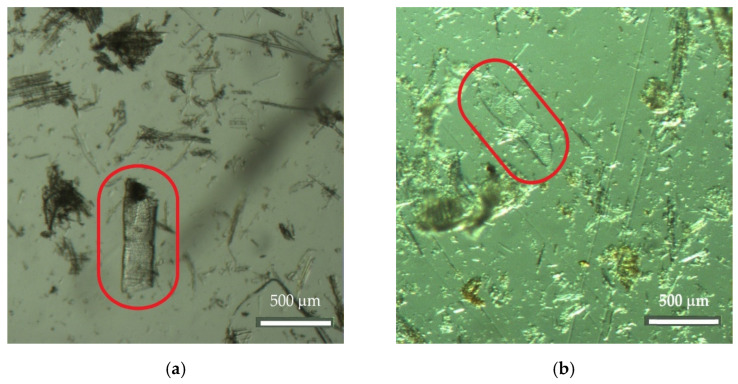
Vessel elements ((**a**) Nest003 and (**b**) Nest006) (Zoom 20×).

The next step consisted of observing the dissociated material with a binocular magnifying glass, which confirmed the presence of cellular elements of agricultural origin and woody material from softwood and hardwood species as well as from leaves. An example of a plant material of agricultural origin (grasses) is shown in Figure 5, and examples of parenchyma cells from a leaf are shown in Figure 6a–c. Regarding the woody material, Figure 7 shows a tracheid with bordered pits, a cellular structure that occurs exclusively in softwood species with the dual function of providing support and transport, and Figure 8a,b shows examples of vessel elements, cellular structures that serve a transport function and that only occur in hardwood species.

**Figure 9:**
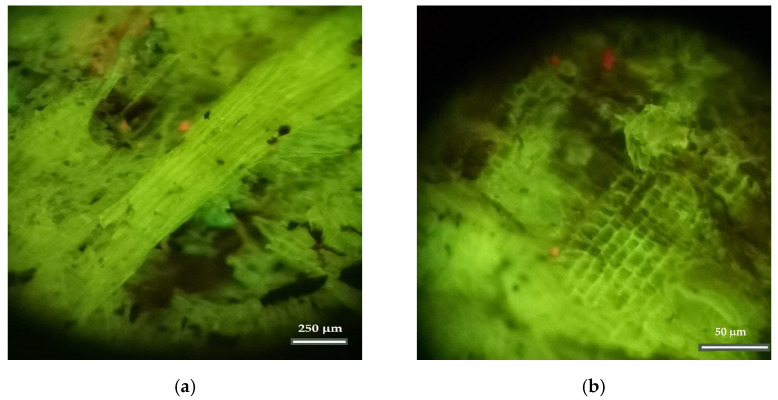
Observations (Nest006) obtained under a fluorescence microscope: (**a**) image of a small flake of non-dissociated chip wood (Zoom 40×); and (**b**) image of a cross-field area between a fiber bundle with a medullary radius with the erect marginal cells of a hardwood (angiosperms) species (Zoom 200×).

**Figure 10:**
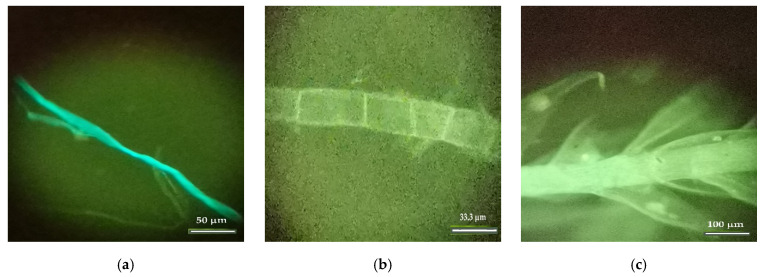
Observations (Nest006) under a fluorescence microscope: (**a**) detail of a hardwood fiber (Zoom 200×); (**b**) a series of axial parenchyma cells (Zoom 300×); and (**c**) details of a structure of a material of agricultural origin (grasses) (Zoom 100×).

The observations that were under a fluorescence microscope confirmed the previous results, as presented in Figure 9 and Figure 10.

Figure 10a shows the details of a hardwood fiber, and Figure 10b shows a series of axial parenchyma cells, which frequently occur in hardwood species (angiosperms) and that serve the function of storing reserve substances in the tree form. Finally, Figure 10c shows a detailed structure of a material of agricultural origin (grasses).

Concerning the difference between the beige and brown strips, it was observed that the difference in color was due to the type of material used. While the beige strips (RGB (197,161,99) CIELab (68.21, 5.43, 37.11)) were formed almost exclusively by woody softwood cells, in the brown strips (RGB (114,82,37) CIELab (37.38, 8.21, 31.04)), materials of different origin are used, such as hardwood cells, leaf tissues, and grasses.

### 3.3. Chemical Analysis

#### 3.3.1. X-ray Diffraction (XRD)

**Figure 11:**
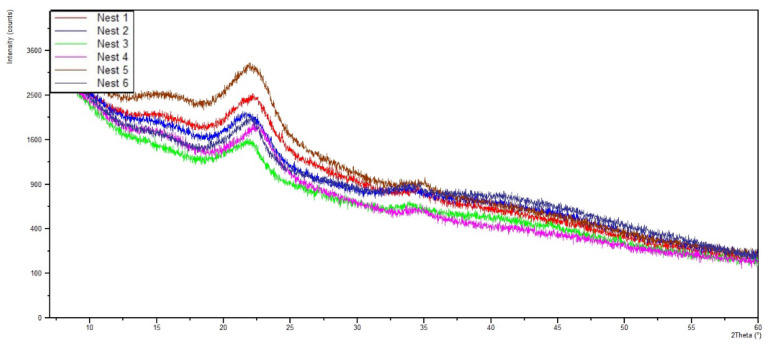
Diffractograms for each of the nests.

**Figure 12:**
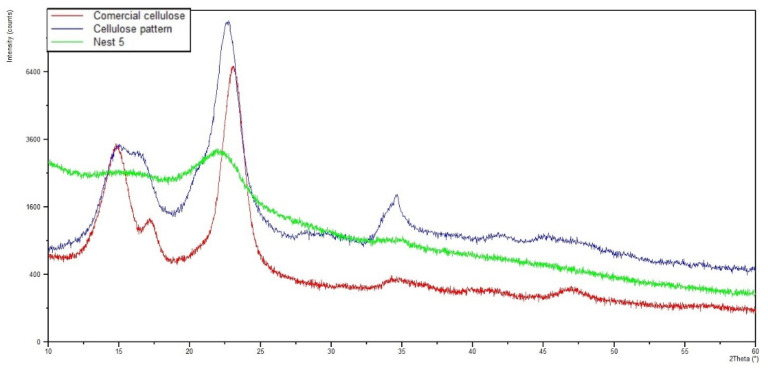
Superposition of the diffractogram of Nest005 with a cellulose pattern and a commercial cellulose sample.

The chemical analysis performed using X-ray diffraction was carried out to identify the origin of the materials used during particle aggregation, namely to determine whether they were of mineral or organic origin. Figure 11 shows the diffractograms for each of the nests, and Figure 12 shows the superposition of the Nest005 diffractogram with a cellulose pattern and a commercial cellulose sample. From this information, it can be stated that the main chemical constituent of the nests is cellulose, which is represented in the diffractograms with a high intensity X-ray diffraction peak in the position from 20 to 25° (2 Theta). The achieved results do not reveal the presence of any mineral material in any of the studied nests, which can be confirmed by the absence of peaks other than those that are characteristics of cellulose. Thus, it was possible to verify that, during nest construction, yellow-legged hornets only use organic materials, such as particles belonging to forest and agricultural species that are agglomerated using the sugars secreted by the hornets themselves.

#### 3.3.2. Scanning Electron Microscopy (SEM) Analysis with EDS

The last stage of the work consisted of using scanning electron microscopy (SEM) to evaluate the chemical elements present in the outer envelope of the nests.

**Figure 13:**
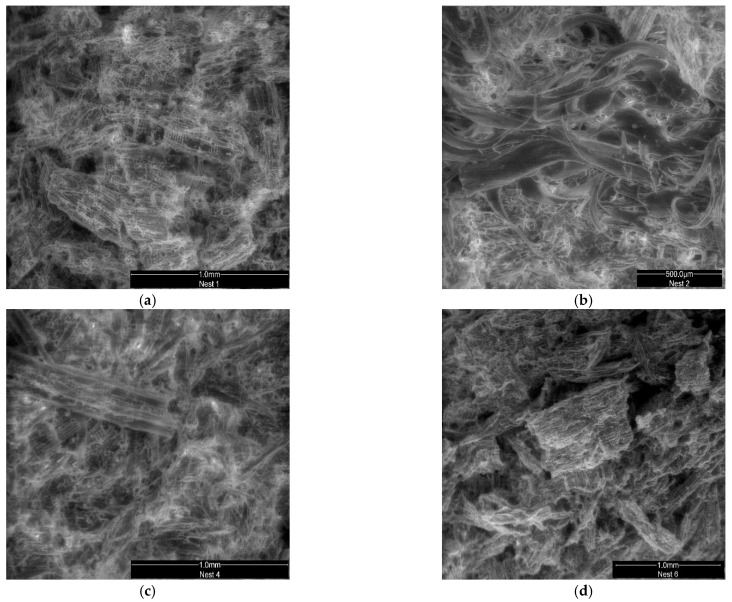
Scanning electron microscopy (SEM) analysis with EDS: (**a**) Nest001, (**b**) Nest002, (**c**) Nest004, and (**d**) Nest006).

**Figure 14:**
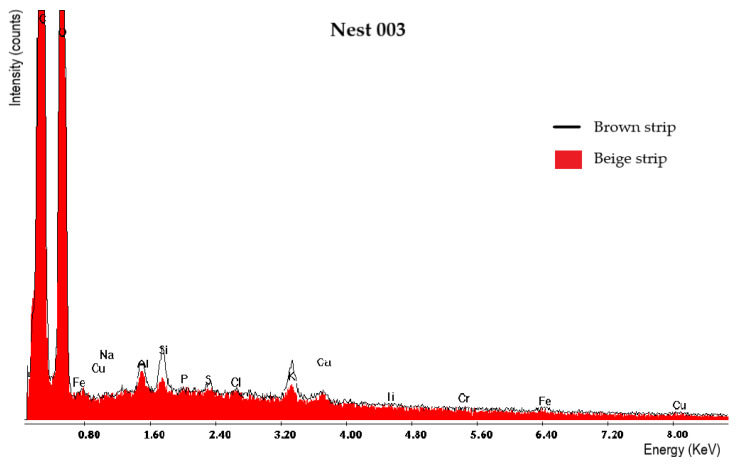
Comparison of the elements in the beige and brown strips of nest Nest003.

Figure 13 shows several photos that were taken by SEM/EDS after the chemical analysis. Table 3 presents the results of the analyses of three nests that were performed separately for the beige and brown strips, which are also graphically presented in Figure 14. As the results that were for the remaining nests were very similar, only these data are shown.

After analyzing the achieved results, it was possible to verify that the main chemical elements present in the nests of yellow-legged hornets are C and O, representing approximately 99% the nests’ composition. These elements are present in the formation of the macromolecules in lignocellulosic materials (cellulose, hemicellulose, and lignin). In addition to these elements, the presence of other microelements, such as Na, Al, Si, K, and Ca, was also observed, although in very small amounts, with values between 0.04% and 0.19%. These small amounts are the reason why they were not identified in the previous chemical analysis. Furthermore, although the nests were built by superimposing parallel beige and brown alternating stripes, it was found that their chemical composition is very similar. In fact, it was found that the differences in the average values of the chemical elements between the beige and brown strips are not statistically significant (*p* < 0.05) for all elements, with the exception of the Si content, whose average content in the beige strips is statistically lower than that of the brown strips (0.07% and 0.19%, respectively) (Table 3). As such, the differences in the color of the strips are due to other factors and not to their chemical composition.

Thus, we can conclude that in the construction of their nests, the *Vespa velutina nigrithorax* only uses materials of plant origin, and no inorganic materials are used to bind the particles.

## 4. Discussion

The *Vespa velutina nigrithorax* (yellow-legged hornet) nests that were analyzed in this study were found at altitudinal ranges between 350–1050 m (Table 1) in forested areas, and four of them were within the ranges that have been established as being normal at altitudes of 200–800 m [38].As indicated by this author, nests can be built at lower and higher altitudes, as is the case for Nest 001 and 002, which were found at altitudes of 1050 m and 1000 m, respectively. These altitude ranges are approximate to those noticed in Italy in 2016 [39].

Almost all of nests were located at the tops of trees, which is true for 73% of the of nests registered in Portugal. One nest, Nest002, was located inside an building, the third most used place by hornets to build their nests. [15]. The five nests located on the treetops were located in four different tree species; nests 5 and 6 were in trees that were of the same species (*Quercus robur*) and were close to each other.

According to [3], in Taiwan, the largest secondary nests of *Vespa velutina nigrithorax* can reach 100 cm in length and 50 cm in diameter. Similar results were found in South Korea by [40], which states that, in November, the nests can reach heights of 90 cm and diameters of 70 cm, but that lower values are visible in August/September (50 cm and 30 cm, respectively). Comparing these results for the native range of *Vespa velutina nigrithorax* with those obtained in northern Portugal, we can conclude that Portuguese nests are smaller since the largest heights only reach 65 cm in November/December and 41 cm in September and 45 cm in diameter November/December and 39 cm in diameter in September. Moreover, although the nests in Portugal are smaller, they have a greater number of combs (4 to 9) in their vertical structure compared to the nests produced in South Korea (4 to 7 combs) [40], i.e., they have a greater number of combs despite their smaller nest volume.

After verification through the macroscopic analysis of samples of *Vespa velutina nigrithorax* nests, which are mainly constituted by small flakes of woody material, the microscopic analysis revealed that during their construction, not only woody materials of softwood (gymnosperms) and hardwood (angiosperms) species, but also leaves and materials of agricultural origin (grasses) are used.

For example, in Figure 4 and Figure 7, a longitudinal tracheid representing about 90–95% of the softwood volume is shown. This type of cells, which have the functions of transport and support in the tree, are exclusive to softwoods and have an average length of about 3 to 5 mm, diameters that range from 0.02 to 0.04 mm on average, and many embroidered holes that serve as communication passages between neighboring tracheids [41,42]. On the other hand, an example of a vessel element is shown in Figure 8. Specialized conducting cells known as vessel elements occur in a significant volume in most hardwoods but are never part of the softwood xylem. Compared to tracheids, vessel elements are short, ranging from 0.2 to 1.0 mm in length and from 0.005 to 0.5 mm in diameter on average [41,42].

Similar conclusions regarding the use of woody material (xylem from rotting wood or from dead parts of living trees) in the construction of *Vespa velutina nigrithorax* nests were also cited by [24,25,43]. According to these authors, *Vespa velutina nigrithorax* uses different fibrous woody materials, mixing them with water and saliva to transform them into a pulpy form to build their nests.

In turn, [44] reported that *V. velutina* build their outer envelope nests using fiber and wood pulp collected from different sources, which is then chewed and mixed with saliva, but detailed information on the sources of material used in the nests was not provided. According to the same authors [44], a slight “varnish” was also observed in the outer layer of some nests, which probably improves their resistance to the adversities of the outside environment. Previous authors claim that *Polistes* wasps (also tropical and social wasp species, just as is *Vespa velutina nigrithorax*) use saliva and other organic secretions to aggregate plant chips [45]. Although the use of woody material in the construction of *V. velutina nigrithorax* nests is frequent, no specific plant resource has yet been identified. In the present study, several examples of images of cellular structures of agricultural origin are shown in Figure 3, Figure 5 and Figure 13. In addition, cross-sectional examples of palisade parenchyma cells of mesophyll, which is the site of photosynthesis, are also shown in Figure 6. Palisade mesophylls are formed by small columnar cells with spaces between the cells and only occur in the leaf structure [46,47,48].

Additionally, chemical analyses were also carried out to find out if, in addition to the lignocellulosic materials identified in the previous analyses, some inorganic materials were also used. For example, in a work on the construction of *V. velutina* nests carried out in Pakistan, [35] refers to the presence of several inorganic elements, such as Ca, K, and Al. However, in the present study, only materials of organic origin were identified, most of which were cellulose. The elemental chemical analysis shows the presence of C and O, which represent approximately 99% of the composition. Identical results are presented by [36], which indicates that during nest construction, *Vespa velutina nigrithorax* mostly used organic products, namely hydrocarbons.

The amounts of other microelements (Na, Al, Si, P, S, K, and Ca) were very low, with values between 0.04% and 0.19%. However, these same microelements are normally present in the vast majority of woody species and in quantities identical to those observed in the cases under study [49,50], which allows us to infer that these microelements come from the woody cellulosic materials that are used in the construction of the nests, as previously mentioned (woody, agricultural materials and leaves). Similar results are also reported by [35] in a study that was also carried out in Pakistan with *V. velutina*. Given these results, we can then infer that this hornet species has the ability to change the type/origin of the materials it uses in the construction of its nests depending on their availability in the region.

## 5. Conclusions

Regardless of the geographic location of the nests and their altitude as well as the height of the support (tree or building), all of the nests had similar structures and compositions.

The results that were obtained here allow us to state that the nests are formed by wood cellulosic materials, that come from surrounding softwood (gymnosperms) and hardwood (angiosperms) species, leftover leaves, and even materials of agricultural origin. The beige strips were formed almost exclusively by woody softwood cells, and the brown strips were primarily formed with hardwood cells, leaf tissues, and grasses.

The main component of the nests was cellulose, which came from these lignocellulosic materials. The elements C and O represent more than 99% of the chemical structure, and no significant mineral components were identified.

In general, the differences in the chemical composition of the beige and brown strips are not statistically significant and are at the probability level of 95%.

Thus, we can conclude that during the next construction, *Vespa velutina nigrithorax* will only use organic materials (particles from forest and agricultural species). These vegetal materials are glued together by organic materials secreted by the hornets, and no significant inorganic materials can be detected.

## Figures and Tables

**Table 1:** *Vespa velutina nigrithorax* nest dimensions.

Nest Number	Nest Place	Lgt_N_	Circ_N_	Wth_N_	Numb_NC_
001	Tree	21.0	60.3	19.0	4.0
002	Indoor	19.2	52.0	18.7	5.0
003	Tree	41.2	58.0	39.0	5.0
004	Tree	46.0	110.0	37.5	7.0
005	Tree	47.7	95.5	33.0	7.0
006	Tree	65.0	134.0	45.0	9.0

Note: Lgt_N_ = overall length in centimeters; Circ_N_ = nest circumference in centimeters; Wth_N_ = width in centimeters; and Numb_NC_ = number of combs.

**Table 2:** *Vespa velutina nigrithorax* nest collection.

Nest Number	Date Removed Nest	Municipality	Nest Place	Tree’s Species Support	H_N_ (m)	Long	Lat	Altitude (m)
001	13/09/2019	Montalegre	Tree	*Pseudotsuga menziesii*	20	−7.785705	41.822405	1050
002	13/09/2019	Montalegre	Indoor		4	−7.789125	41.826872	1000
003	20/09/2019	Vila Real	Tree	*Pinus pinaster*	24	−7.741917	41.287344	500
004	04/11/2019	Montalegre	Tree	*Fraxinus* *angustifolia*	17	−8.032647	41.723639	350
005	06/11/2019	Montalegre	Tree	*Quercus robur*	15	−7.948567	41.638379	850
006	13/12/2019	Montalegre	Tree	*Quercus robur*	18	−7.981200	41.674153	700

Note: H_N_ = height of the nest from the ground in meters.

**Table 3:** Results of the nest chemical composition by strip and its corresponding means comparison test.

Elements	Average			Tukey Test	
	All ± sd	Beige	Brown	Sig.	*p*-Value
C	63.01 ± 1.31	62.41	63.61	ns	0.1165
O	36.35 ± 1.36	37.00	35.70	ns	0.0977
Na	0.08 ± 0.02	0.08	0.08	ns	0.7961
Al	0.19 ± 0.07	0.22	0.17	ns	0.2094
Si	0.13 ± 0.07	0.07	0.19	***	<0.0001
P	0.04 ± 0.02	0.04	0.04	ns	0.7618
S	0.04 ± 0.02	0.04	0.05	ns	0.0588
Cl	0.01 ± 0.01	0.02	0.01	ns	0.0959
K	0.05 ± 0.02	0.04	0.05	ns	0.3913
Ca	0.04 ± 0.02	0.04	0.05	ns	0.1256
Ti	0.01 ± 0.00	0.01	0.01	ns	1.0000
Cr	0.01 ± 0.01	0.01	0.00	ns	0.0734
Fe	0.02 ± 0.01	0.02	0.02	ns	0.5701
Cu	0.02 ± 0.01	0.02	0.02	ns	0.3628

Note: sd = standard desviation; Sig. = significance to 95%, ns-not significant, ***-highly significant.

## Data Availability

Not applicable.
